# Dynamic Hip Screw with Trochanter-Stabilizing Plate Compared with Proximal Femoral Nail Antirotation as a Treatment for Unstable AO/OTA 31-A2 and 31-A3 Intertrochanteric Fractures

**DOI:** 10.1155/2020/1896935

**Published:** 2020-08-18

**Authors:** Chun-Wei Fu, Ji-Ying Chen, Yueh-Ching Liu, Kuang-Wen Liao, Yung-Chang Lu

**Affiliations:** ^1^Department of Orthopedic Surgery, Mackay Memorial Hospital, Taipei, Taiwan; ^2^Department of Biological Science and Technology National Chiao Tung University, Hsinchu, Taiwan; ^3^Institute of Molecular Medicine and Bioengineering, National Chiao Tung University, Hsinchu, Taiwan

## Abstract

**Background:**

The dynamic hip screw (DHS) with the addition of an angular stable trochanter-stabilizing plate (TSP) has been considered the ideal treatment for the unstable intertrochanteric fracture type. However, there have been few comparisons between DHS+TSP augmentation with intramedullary (IM) nailing. The aim of this retrospectively registered study was to compare the clinical outcomes of patients with the unstable type of intertrochanteric fractures treated with DHS+TSP or IM nailing (proximal femoral nail antirotation (PFNA)).

**Methods:**

From June 2013 to April 2018, 358 patients with proximal femur fracture AO/OTA type 31A2 and 31A3 treated with PFNA or DHS+TSP and followed for ≥10 months postoperatively were included. The surgical-dependent outcome evaluation included the operation time, intraoperative blood loss, postoperative decrease in hemoglobin, and blood transfusion amount. Functional status was also measured. Radiographic findings and postoperative complications were recorded and analyzed.

**Result:**

The operation time was significantly shorter in the DHS+TSP group than that in the PFNA group for both A2 and A3 fractures (A2 type: 84.0 vs.96.4 min; *p* < 0.05; A3 type: 102.4 vs.116.1 min; *p* < 0.05). Postoperative decrease in hemoglobin was more significant in the PFNA group than that in the DHS+TSP group for both fracture types (A2 type: −1.88 vs. −1.29 (mg/dL); *p* < 0.05; A3 type: −1.63 vs. −1.04 (mg/dL); *p* < 0.05). However, the patients treated with DHS+TSP had significantly more residual pain than those treated with PFNA during the final follow-up (Visual Analog Scale score, A2 type: 28.4 vs.23.2; *p* < 0.05; A3 type: 27.5 vs.23.6; *p* < 0.05) and complained of greater implant irritation.

**Conclusion:**

We found that DHS+TSP was associated with less operation time and less postoperative decrease in hemoglobin but more residual pain and implant irritation than those of PFNA. As a treatment for unstable intertrochanteric fracture, DHS+TSP provided ideal surgical outcomes which were not inferior to the PFNA.

## 1. Introduction

Proximal femur fractures have increased with increasing mean population age and have become a public health issue. There is no consensus on whether extramedullary or intramedullary (IM) fixation is the best treatment for extracapsular fractures. From a biomechanical perspective, IM nail fixation can resist higher loading forces [1] and may provide greater stability than that of extramedullary fixation for unstable fracture patterns, including posteromedial wall involvement, insufficient lateral wall thickness, and reverse oblique type [2–5].

Nevertheless, many studies have reported that the dynamic hip screw (DHS) was not inferior to IM nailing even for the unstable type of intertrochanteric fracture [6–9]. Furthermore, addition of an angularly stable trochanter-stabilizing plate (TSP) with locking screws has been used to reinforce DHS fixation and reduce medialization and shortening of the femoral shaft and is regarded as a solution for treating unstable type fractures that use a more familiar approach and implant application [5, 10–13].

The indications for IM nailing or DHS+TSP augmentation overlap, but few studies have compared these two types of implants for treating the unstable type of intertrochanteric fractures. The aim of this retrospective study was to compare the clinical outcomes of patients with the unstable type of intertrochanteric fractures treated with IM nailing (proximal femoral nail antirotation (PFNA))or DHS with TSP (DHS+TSP) with regard to operation time, blood loss, pain relief, functional outcome, osseous union rate, and implant-related complications.

## 2. Materials and Methods

From June 2013 to April 2018, the patients with AO/OTA type 31A2 and 31A3 proximal femur fractures treated with DHS+TSP augmentation or with PFNA who had been followed ≥10 months were considered for inclusion. The exclusion criteria were (1) pathological fractures, (2) patients with multiple traumas, and (3) periprosthetic or peri-implant fractures. The operations were performed with the patients under spinal or general anesthesia in the supine position on a fracture table. The patients' radiographic results were presented and discussed preoperatively in the daily morning conference by our orthopedic surgeons to categorize the fracture pattern and suggest the proper implant for fixation. In the consensus of our faculties, once the fracture was categorized as an unstable intertrochanteric fracture type, TSP augmentation in addition to DHS fixation was indicated; otherwise, PFNA was chosen. The application of DHS+TSP or PFNA was decided finally by the orthopedic surgeon according to their respective experience.

For surgical-dependent outcome evaluation, the operation time, intraoperative blood loss, postoperative decrease in hemoglobin (12  hours postoperative level minus preoperative level), and amount of blood transfusion was recorded. The pain from the operated side was recorded at the first outpatient clinic visit after surgery using the Visual Analog Scale (VAS) score (0 indicates no pain, and 100 indicates intolerable pain). Implant-related irritation was recorded as the patients' subjective complaint of a foreign sensation at the surgical site. For functional outcome evaluation, we used the EuroQoL-5D (EQ-5D) questionnaire to evaluate the patients' quality of life and functional status preoperatively and at the last follow-up. The EQ-5D questionnaire assesses mobility, self-care ability, level of activities, pain/discomfort, and anxiety/depression. Each of the dimensions was assigned one of three levels (no problems, some problems, and severe problems). The EQ-5D index score is calculated from these answers and gives a maximum score of 1.0, which indicates a very good quality of life, and the lowest is a score of 0, which is equivalent to death [14]. In the radiographic outcome evaluation, we categorized fracture nonunion (defined as failure of osseous union at the end of 9 months or no signs of bone healing for three consecutive months) and implant-related complications (screw cutout and peri-implant fracture) as “failure of osteosynthesis.” Any kind of secondary surgery was considered to be a reoperation and recorded according to the type of reoperation performed.

## 3. Statistical Analyses

The data are presented as the mean and standard deviation for continuous variables or as numbers and percentages for categorical variables. All data were entered and analyzed in SPSS software (version 17.0; SPSS Inc., Chicago, IL). The independent-test was used for continuous outcome variables analysis, and the Pearson chi-square test was used for categorical outcome variables analysis. A *p* value of < 0.05 indicated statistical significance.

## 4. Results

The patients' characteristics are presented in Table 1. For A2 fractures, 171 patients underwent DHS+TPS fixation and 70 patients underwent PFNA fixation. The operation time was significantly shorter in the DHS+TSP group than that in the PFNA group (84.0 vs. 96.4 min, respectively; *p* < 0.05) (Table 2). Although there were no significant between-group differences in intraoperative blood loss and blood replacement, less postoperative decrease in hemoglobin was noted in the DHS+TSP group (−1.29 vs. −1.88 mg/dL, *p* < 0.05). In postoperative follow-up, no significant between-group difference was noted in the EQ-5D index score and functional status changes. We separately assessed the patient's mobility status according to the “Mobility” dimension of the EQ-5D questionnaire. We found that both groups of patients suffered from deterioration of their mobility status after surgery, but no significant between-group difference was noted (Table 3). However, a higher postoperative VAS score (28.4 vs. 23.2, *p* < 0.05) was noted in the DHS+TSP fixation group, which indicated that this group suffered from more residual pain. Additionally, more patients in the DHS+TSP group than those in the PFNA group complained of an implant-related irritation (*p* < 0.05).

In the postoperative radiographic evaluation, 94.2% of the DHS+TSP group and 94.3% of the PFNA group reached osseous union without implant failure. Ten patients in the DHS+TSP group suffered from failure of osteosynthesis, which included nine screw cutouts and one fracture nonunion. In the PFNA group, three patients suffered from blade screw cutout and one fracture nonunion. No between-group differences in the fracture union rate (*p* = 0.627), failure of osteosynthesis rate (*p* = 0.967), and reoperations (*p* = 0.798) were noted (Table 4).

Similar results were noted for the patients with the A3 type fracture. Compared with DHS+TSP, PFNA fixation had a significantly longer operation time (102.4 vs. 116.1 min, *p* < 0.05) and a greater postoperative decrease in hemoglobin (−1.04 vs. −1.63 mg/dL, *p* < 0.05). Similarly, the patients in the DHS+TSP group suffered from more residual pain (*p* < 0.05) than those in the PFNA fixation group, but there was no significant difference in implant irritation (*p* = 0.835). In postoperative follow-up, no significant in between-group differences in the change in EQ-5D index score (Table 5) or mobility status were noted (Table 6). Both types of implant fixation achieved ideal osseous union, and the rates of implant-related complications were comparable (Table 7).

**Figure 1:**
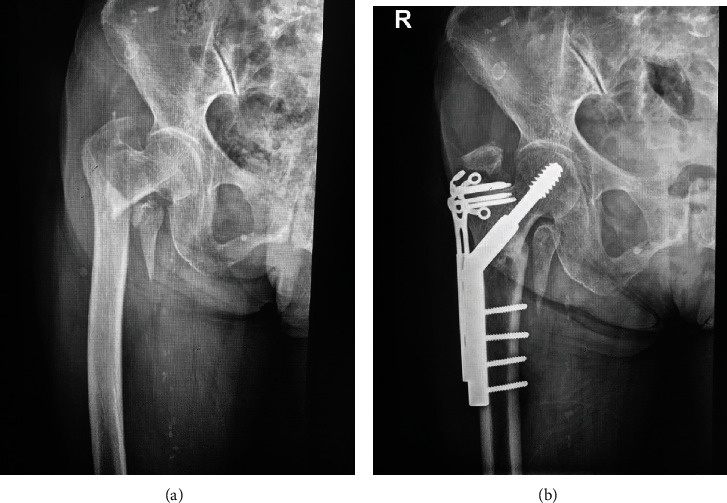
Preoperative and postoperative anteroposterior plain radiographs of a 31A2 fracture treated with DHS+TSP. (a) Preoperative and (b) postoperative anteroposterior plain radiographs of a 31A2 fracture treated with dynamic hip screw + trochanter-stabilizing plate. (b) Postoperative radiographs showing osseous union over the fracture site but greater trochanteric tip avulsion fractures.

In the DHS+TSP group, 11 patients (nine with A2 type fractures and two with A3 type fractures) showed greater trochanteric tip avulsion fracture or bone absorption in follow-up radiography (Figure 1). A comparison of this group of patients (*n* = 11) with the other patients who received DHS+TSP fixation without associated greater trochanteric tip fracture (*n* = 223) showed no significant difference in clinical outcomes by VAS scores (24.5 ± 5.2 vs. 28.2 ± 0.4, respectively; *p* = 0.414) and EQ-5D index scores (0.55 ± 0.12 vs. 0.59 ± 0.14, respectively; *p* = 0.561) at the last follow-up, and only two of 11 patients mentioned an implant irritation complaint.

## 5. Discussion

In this retrospective study comparing DHS+TSP fixation with PFNA fixation of unstable type and reverse oblique type (AO/OTA 31A2 and 31A3) intertrochanteric fractures, the patients who received DHS+TSP fixation had significantly shorter operation time and less postoperative decrease in hemoglobin than those who received PFNA fixation, but the DHS+TSP group had more residual pain and implant irritation complaints. However, there were no significant differences in the amount of blood transfusion, quality of life, osseous union rate, and failure of osteosynthesis rate between these treatment types.

Previous systemic reviews have found that IM nailing was associated with shorter operation time and less intraoperative blood loss [15, 16]. There are several reasons for the inconsistency between our results and those of the previous studies. First, we separately analyzed the unstable type and reverse oblique intertrochanteric fractures, which differed from the previous studies that treated both stable and unstable type fractures. The unstable type fracture often leads to difficulty in closed fracture reduction. Once the closed reduction fails, it requires conversion to open reduction or experienced reduction technique. The change in the surgical plan may have elongated the surgical time, especially when closed nailing was initially planned. In other words, IM nailing in unstable type fractures requires greater surgical technique skill [17]. Second, for unstable fracture fixation, a long nail was chosen sometimes to achieve adequate implant working length, which often increased the operation time for nail and distal screw application [18, 19].

When assessing for blood loss, no significant differences were found in the intraoperative blood loss and amount of blood transfusion between the two fixation types, but the PFNA group had a greater change in postoperative hemoglobin level relative to the preoperative level. These results also differed from those in the published studies in which the use of an IM nail resulted in less blood loss [15, 16]. These differences in results may be related to the longer operation time in managing unstable type fractures treated with PFNA. Assessing the blood loss indirectly by measuring the change in hemoglobin levels also may have given an opposite result from those of previous studies because “internal” blood loss after IM nailing was not assessed in previous studies [8]. Although greater change of hemoglobin levels was noted in the PFNA group, no direct correlation was found between hemoglobin levels and blood transfusion. Therefore, some probable reasons might explain why the decrease of hemoglobin levels was not correlated to the transfusion amounts between the two groups. First, the baseline hemoglobin level of the patients was not exactly the same between both groups (In 31A2 fracture, the hemoglobin level in the DHS+TSP group is 11.41 mg/dL while in the PFNA group, 11.92 mg/dL. In 31A3 fracture, the hemoglobin level in the DHS+TSP group is 11.17 mg/dL while in the PFNA group, 11.94 mg/dL.). In our department, hemoglobin of 9 mg/dL was adapted as the blood transfusion threshold for patients accepted major surgery. Besides, several factors might influence the decision for blood transfusion, such as the patient's underlying problem and immediate medical condition, blood loss during operation, and amount of drainage. Thus, hemoglobin level was only one of the several parameters to determine whether or not blood transfusion should be done.

In the postoperative follow-up comparison, no significant between-group difference in the quality of life measured by the EQ-5D was found. However, we did find that the group of patients who received DHS+TSP fixation had a higher pain score in the clinical follow-up (residual pain) and more complaints of implant irritation. It is reasonable that an additional plate fixation over the greater trochanter would lead to greater irritation between soft tissue and the implant, but no previous studies have discussed this aspect of DHS+TSP fixation.

A total of 11 patients (nine with A2 type fractures and two with A3 type fractures) with TSP fixation had greater trochanter tip avulsion fractures or absorption in the follow-up radiography. We thought that these associated avulsion fractures were caused by multiple screws cutting through the greater trochanteric tip and subsequent displacement of bone caused by adjacent muscle contraction. However, these tip fractures were simply radiographical findings, and they did not cause a decrease in the patients' quality of life or increase their pain.

In our study, the osteosynthesis failure rate for the A2 type fracture was 5.8% (10/171) in the DHS+TSP group and 5.7% (4/70) in the PFNA group and for the A3 type fracture was 1.6% (1/63) in the DHS+TSP group and 7.4% (4/54) in the PFNA group. However, not all of these complications result in reoperation because elderly patients commonly have a deteriorating physical condition that makes them unsuitable for a second surgery or they are unwilling. In a further analysis of the overall implant-related complications of both implants, we found that there was a higher proportion of lag screw cutout in the DHS+TSP group (10 of 12) than that in the PFNA group (3 of 8) even though the difference was not statistically significant. A screw tip–apex distance (TAD) >25 mm [20] may be an indicator of further lag screw cutout, but inadequate TAD was noted only in three patients in the DHS+TSP group and in one patient in the PFNA group, which suggests that some other factors may affect the screw cutout. Another explanation of this result is that the blade screw of the PFNA has the advantage of fitting via bone compaction and requires less bone removal, which prevents screw cutout [21, 22]. Additionally, all of the nonunion patients in the PFNA group had varying degrees of varus reduction initially, which caused malpositioning of the implant and led to further nonunion or implant breakage [23, 24]. Published studies have stated that the abovementioned complications were mostly caused by surgical imperfection or improper technique rather than implant selection [8, 24].

Further, identifying the thickness of the proximal femur lateral wall is important, which determined the implant selection and patient's outcome. Several literatures have stated that TSP proved an additional buttress force and was an ideal treatment for those patients with insufficient lateral wall thickness of the greater trochanter [4, 12]. Based on the current AO/OTA classification, patients with lateral wall thickness of <20.5 mm in the group of 31A2 (unstable type intertrochanteric fracture) were included. However, most of the patients with insufficient lateral wall thickness were also found to be associated with an intermediate fragment (classified as 31A2.2). Only 40 (40/171) and 11 patients (11/70) in the DHS+TSP group and the PFNA group, respectively, were identified as pure lateral wall insufficient (which only meet the criteria of lateral wall thickness < 20.5 mm, without intermediate fragment). Subgroup analysis was not performed since this subgroup has a relatively small number of patients. Both DHS+TSP and PFNA were thought to serve as a primary choice for treating patients who are lateral wall insufficient with intertrochanteric fracture. However, to determine which one is superior to another, more patients who are pure lateral wall insufficient with intertrochanteric fracture must be enrolled.

## 6. Study Limitations

Several limitations in our study should be considered. First, this was a retrospective study, so the studied implants were not randomly selected, which means that the selection involved the surgeon's preference, so selection bias could have occurred. Second, the patients' underlying problem (e.g., osteoporosis) and comorbidities during hospitalization were not documented in detail; each patient's general condition was assessed only by their American Society of Anesthesiologists score and mobility status. Additionally, patients with insufficient follow-up or who dropped out during the follow-up period were not included in this study. All of these factors may have influenced the identification of the osseous union and affected the assessment of complications. Third, we also could not clearly document when the patient started weight bearing even though some studies regard the time to weight bearing as an important parameter for evaluating the stability of implant fixation [25, 26]. A comparison of surgical incision length between the two implants was also not performed in our study. With the trend of pursuing minimally invasive osteosynthesis, the size of the surgical wound may be a concern when choosing an implant.

## 7. Conclusion

To the best of our knowledge, this is the first study to compare outcomes of treating unstable intertrochanteric fractures (AO/OTA 31A2 and 31A3) with IM nailing (PFNA) or with DHS+TSP augmentation. To better understand the basis for indications for either IM nailing or DHS+TSP, we separately analyzed the outcomes according to the different types of unstable intertrochanteric fractures. In this retrospective study, we found that DHS+TSP augmentation and PFNA fixation were both able to provide good clinical results for the treatment of AO/OTA 31A2 and 31A3 intertrochanteric fractures. Compared with PFNA, DHS+TSP was associated with shorter operation time and less postoperative decrease in hemoglobin levels without significantly increasing intraoperative blood loss and the amount of blood transfusion. However, because of the additional plate set on the greater trochanter, TSP inevitably caused greater residual pain and a greater number of implant irritation complaints. We concluded that DHS+TSP provided ideal surgical outcomes, which were not inferior to the PFNA in treatment for unstable AO/OTA 31A2 and 31A3 intertrochanteric fracture.

## Figures and Tables

**Table 1:** Patient demographics.

	DHS+TSP	PFNA^a^
Total number (*n* = 358)	234	124
Mean age, years (range)	79.6 (35–97)	77.4 (26–95)
Sex (*n* = 358)		
Female (%)	160 (65.8%)	72 (58.1%)
ASA classification^b^ (*n* = 358)		
I (%)	10 (4%)	11 (8.8%)
II (%)	36 (15.4%)	39 (31.5%)
III (%)	188 (80.3%)	74 (59.7%)
Fracture type (AO/OTA) (*n* = 358)		
31A2	171	70
31A3	63	54
Mean follow-up times, months (range)	13.1(10–17)	12.9(10–18)

^a^Including short and long PFNA, long nail percentage: 23% (28/124). ^b^American Society of Anesthesiologists classification of comorbidities.

**Table 2:** Surgical-dependent and patient-related outcomes in 31A2 type fractures.

	DHS+TSP (*n* = 171)	PFNA (*n* = 70)	*p* value
Operation time, mean ± SD, mins	84.0 ± 27.1	96.4 ± 49.4	0.013
Intraoperative blood loss, mean ± SD, mL	233.6 ± 95.4	214.1 ± 146.3	0.225
∗postoperative decrease in hemoglobin, mean ± SD, mg/dL	−1.29 ± 1.45	−1.88 ± 1.20	0.003
Transfusion, mean ± SD, units	0.9 ± 1.2	0.9 ± 1.8	0.784
EQ-5D, mean ± SD			
Preoperative	0.69 ± 0.11	0.71 ± 0.09	0.272
Final follow-up	0.59 ± 0.14	0.61 ± 0.10	0.211
VAS, mean ± SD			
First postoperative clinic follow-up	47.2 ± 8.7	45.8 ± 5.7	0.213
Final follow-up	28.4 ± 7.1	23.2 ± 4.7	<0.001

Note: data presented as mean ± standard deviation unless otherwise indicated. ^∗^Decrease between the preoperative hemoglobin and 12 h postoperative hemoglobin levels.

**Table 3:** Patient's mobility status (from EQ-5D questionnaire) in 31A2 fractures.

	Implant	No problem	Some problem	Bedridden	Total	*p* value
Preoperative	DHS+TSP, *n* (%)	113 (66)	50 (29)	8 (5)	171	
	PFNA, *n* (%)	48 (68)	18 (26)	4 (6)	70	0.83
Final follow-up	DHS+TSP, *n* (%)	86 (51)	68 (39)	17 (10)	171	
	PFNA, *n* (%)	42 (60)	22 (31)	6 (9)	70	0.386

**Table 4:** Image outcome, implant-related complications, and reoperation in 31A2 fractures.

	DHS+TSP *n* = 171 (%)	PFNA *n* = 70, (%)	*p* value
Osseous union	161 (94.2)	66 (94.3)	0.627
Failure of osteosynthesis			
All	10 (5.8)	4 (5.7)	0.967
Screw cutout	9 (5.3)	3 (4.3)	
Nonunion	1 (0.58)	1 (1.4)	
Implant irritation	26 (15.2)	4 (5.7)	0.043
Reoperation			
All	6 (3.5)	3 (4.3)	0.798
Implant removal	4 (2.3)	0	
New osteosynthesis	2 (1.2)	0	
Bipolar hemiarthroplasty	0	1 (1.4)	
Total hip arthroplasty	0	2 (2.9)	

**Table 5:** Surgical-dependent and patient-related outcomes in 31A3 type fractures.

	DHS+TSP (*n* = 63)	PFNA (*n* = 54)	*p* value
Operation time, mean ± SD, mins	102.4 ± 46.1	116.1 ± 32.1	0.008
Intraoperative blood loss, mean ± SD, mL	323.9 ± 209.2	344.9 ± 187.2	0.57
Postoperative decrease in hemoglobin, mean ± SD, mg/dL	−1.04 ± 1.44	−1.63 ± 1.69	0.04
Transfusion, mean ± SD, units	1.8 ± 2.1	1.7 ± 2.1	0.79
EQ-5D, mean ± SD			
Preoperative	0.69 ± 0.14	0.72 ± 0.11	0.208
Final follow-up	0.59 ± 0.16	0.62 ± 0.12	0.302
VAS, mean ± SD			
First postoperative clinic follow-up	46.0 ± 8.5	44.8 ± 5.4	0.37
Final follow-up	27.5 ± 6.0	23.6 ± 6.2	<0.001

**Table 6:** Patient's mobility status (from EQ-5D questionnaire) in 31A3 fractures.

	Implant	No problem	Some problem	Bedridden	Total	*p* value
Preoperative	DHS+TSP, *n* (%)	44 (69)	18 (29)	1 (2)	63	
	PFNA, *n* (%)	41 (76)	12 (22)	1 (2)	54	0.735
Final follow-up	DHS+TSP, *n* (%)	36 (57)	25 (40)	2 (3)	63	
	PFNA, *n* (%)	36 (67)	17 (31)	1 (2)	54	0.671

**Table 7:** Image outcome, implant-related complications, and reoperation in 31A3 fractures.

	DHS+TSP *n* = 63, (%)	PFNA *n* = 54, (%)	*p* value
Osseous union	61	50	0.303
Failure of osteosynthesis			
All	2 (3.2)	4 (7.4)	0.122
Screw cutout	1 (1.6)	0	
Nonunion	1 (1.6)	3 (5.6)	
Peri-implant fracture	0	1 (1.9)	
Implant irritation	13 (20.6)	12 (22.2)	0.835
Reoperation			
All	6 (9.5)	6 (11.1)	0.779
Implant removal	6 (9.5)	4 (7.4)	
New osteosynthesis	0	2 (3.7)	

## Data Availability

The data used to support the findings of this study are available from the corresponding author upon request
